# Assessment of Changes in the Quality of Voice in Post-thyroidectomy Patients With Intact Recurrent and Superior Laryngeal Nerve Function

**DOI:** 10.7759/cureus.60873

**Published:** 2024-05-22

**Authors:** Anjan K Sahoo, Prasanta K Sahoo, Vikas Gupta, Ganakalyan Behera, Shaila Sidam, Utkal P Mishra, Aparna Chavan, Rashma Binu, Shivam Gour, Dhanoush Kumar Velayutham, Pooja, Twisha Chatterjee, Debrup Pal

**Affiliations:** 1 Otolaryngology - Head and Neck Surgery, All India Institute of Medical Sciences, Bhopal, Bhopal, IND

**Keywords:** laryngeal endoscopy, voice analysis, postoperative outcomes, maximum phonation duration, shimmer intensity, first formant frequency, fundamental frequency, laryngeal nerves, voice change, thyroidectomy

## Abstract

Background

Thyroidectomy is a routinely performed surgical procedure used to treat benign, malignant, and some hormonal disorders of the thyroid that are not responsive to medical therapy. Voice alterations following thyroid surgery are well-documented and often attributed to recurrent laryngeal nerve dysfunction. However, subtle changes in voice quality can persist despite anatomically intact laryngeal nerves. This study aimed to quantify post-thyroidectomy voice changes in patients with intact laryngeal nerves, focusing on fundamental frequency, first formant frequency, shimmer intensity, and maximum phonation duration.

Methodology

This cross-sectional study was conducted at a tertiary referral center in central India and focused on post-thyroidectomy patients with normal vocal cord function. Preoperative assessments included laryngeal endoscopy and voice recording using a computer program, with evaluations repeated at one and three months post-surgery. Patients with normal laryngeal endoscopic findings underwent voice analysis and provided feedback on subjective voice changes. The PRAAT version 6.2 software was utilized for voice analysis.

Results

The study included 41 patients with normal laryngoscopic findings after thyroid surgery, with the majority being female (85.4%) and the average age being 42.4 years. Hemithyroidectomy was performed in 41.4% of patients and total thyroidectomy in 58.6%, with eight patients undergoing central compartment neck dissection. Except for one patient, the majority reported no subjective change in voice following surgery. Objective voice analysis showed statistically significant changes in the one-month postoperative period compared to preoperative values, including a 5.87% decrease in fundamental frequency, a 1.37% decrease in shimmer intensity, and a 6.24% decrease in first formant frequency, along with a 4.35% decrease in maximum phonatory duration. These trends persisted at the three-month postoperative period, although values approached close to preoperative levels. Results revealed statistically significant alterations in voice parameters, particularly fundamental frequency and first formant frequency, with greater values observed in total thyroidectomy patients. Shimmer intensity also exhibited slight changes. Comparison between hemithyroidectomy and total thyroidectomy groups revealed no significant differences in fundamental frequency, first formant frequency, and shimmer. However, maximum phonation duration showed a significantly greater change in the hemithyroidectomy group at both one-month and three-month postoperative intervals.

Conclusions

This study on post-thyroidectomy patients with normal vocal cord movement revealed significant changes in voice parameters postoperatively, with most patients reporting no subjective voice changes. The findings highlight the importance of objective voice analysis in assessing post-thyroidectomy voice outcomes.

## Introduction

Thyroidectomy involves the removal of part or all of the thyroid gland and is one of the most common surgical procedures performed worldwide. While the primary aim of thyroidectomy is to treat thyroid disorders such as goiter, thyroid nodules, or thyroid cancer, it often leads to voice alterations postoperatively. These alterations are multifaceted and can significantly impact patients’ quality of life, particularly those who rely on their voices for professional or personal reasons. Voice alterations following thyroid surgery are primarily attributed to the involvement of the recurrent laryngeal nerve (RLN) and the superior laryngeal nerve (SLN), which innervate the muscles controlling vocal fold movement and tension [1]. The RLN, in particular, is at risk of injury during thyroidectomy due to its close proximity to the thyroid gland. Studies have reported that approximately 5-10% of thyroidectomy cases involve RLN dysfunction, with 5% experiencing temporary changes in voice and 1-3% suffering from permanent voice alterations [2]. The severity and duration of dysphonia associated with RLN dysfunction correlate with the extent of nerve injury. While RLN dysfunction is a well-recognized cause of voice alterations post-thyroidectomy, there is growing recognition that other factors may contribute to changes in voice quality, even in patients with anatomically and functionally intact laryngeal nerves [3]. Some patients exhibit minimal voice alterations postoperatively despite preserved laryngeal nerve function and normal vocal cord movements. These subtle changes in voice can be of concern, particularly for professional voice users such as singers, anchors, and radio jockeys, whose livelihoods depend on vocal performance. However, to date, there is a paucity of comprehensive studies that quantify alterations in voice parameters in post-thyroidectomy patients with intact laryngeal nerves. Our research aims to fill this gap by objectively assessing changes in four essential parameters of voice, namely, fundamental frequency, first formant frequency, shimmer, and maximum phonation duration. These parameters are fundamental aspects of voice production and are commonly used to evaluate vocal function and quality. Fundamental frequency refers to the perceived pitch of a voice and is determined by the rate of vibration of the vocal folds [4]. Changes in fundamental frequency can indicate alterations in vocal fold tension and length, which may occur as a result of thyroid surgery. The first formant frequency represents the resonance frequency of the vocal tract and is influenced by the configuration of the vocal tract [5]. Alterations in first formant frequency may indicate changes in vocal tract shape or size, which could occur due to surgical manipulation or tissue trauma during thyroidectomy. Shimmer is a measure of the variability in the amplitude of vocal fold vibrations and is indicative of voice instability or roughness [6]. Changes in shimmer may reflect subtle disruptions in vocal fold mucosal wave patterns or tissue integrity following thyroid surgery. Meanwhile, maximum phonation duration refers to the length of time a person can sustain a phonation or vocalization [7]. Changes in maximum phonation duration can provide insights into vocal endurance and respiratory support, which may be affected by surgical trauma or postoperative recovery.

This study objectively evaluates these four essential parameters of voice in patients who have undergone thyroidectomy with intact recurrent and superior laryngeal nerve function in the postoperative period. Furthermore, the study aims to evaluate the variations in these voice parameters based on the extent of thyroid surgery by comparing voice parameters between patients undergoing hemithyroidectomy and total thyroidectomy.

## Materials and methods

This cross-sectional study was conducted in the Department of Otorhinolaryngology-Head and Neck Surgery of a tertiary care referral center in central India. Before commencement, this study was reviewed and approved by the Institutional Human Ethics Committee, All India Institute of Medical Sciences, Bhopal, India (approval number: IHEC-LOP/2023/IL0868). Written and informed consent were obtained from all study participants. All post-thyroidectomy patients exhibiting normal vocal cord movement in postoperative laryngeal endoscopy were included in the study. Patients with postoperative vocal cord paresis or palsy were excluded from the study. Patients with a change in voice before surgery were also excluded from the study. A detailed overview of the comprehensive inclusion and exclusion criteria is presented in Table 1.

**Table 1:** Inclusion and exclusion criteria.

Criteria	Description
Inclusion criteria	All patients who have undergone thyroid surgery for benign or malignant lesions of the thyroid exhibiting normal vocal cord movement in postoperative laryngeal endoscopy
Exclusion criteria	Patients with postoperative vocal cord paresis or palsy. Patients with a history of change in voice before surgery. Patients with thyroid carcinoma with pulmonary metastasis. Patients who have undergone laryngectomy or tracheal resection along with thyroidectomy. Patients with anaplastic carcinoma of the thyroid who underwent postoperative radiotherapy to the neck. Patients who were lost to follow-up

The preoperative assessment included laryngeal endoscopy and recording of voice using a computer program. These evaluations were repeated at the one-month and three-month postoperative period. The resting position, symmetry, mobility of vocal cords as well as glottal closure were assessed in laryngeal endoscopic evaluation. Only patients with normal findings in all the above parameters in laryngeal endoscopy underwent voice analysis. Additionally, patients were asked to provide subjective feedback regarding any changes in their voice.

Voice recording

Participants were seated comfortably in a sound-treated booth in front of a sound level meter and microphone. An omnidirectional microphone (MAX CM 903 Electret condenser microphone) was employed for voice recording. To ensure consistency, the sound level meter and microphone were placed at a 45-degree angle from the participant’s mouth, one on each side, with a fixed 6-inch mouth-to-microphone distance. The mouth-to-microphone distance was maintained during all recordings using a measuring device that each participant held against his or her chin. One of the senior authors collected the voice data, while the other author monitored vocalization to ensure appropriate intensity levels. Participants were instructed to sustain the vowels “a,” “e,” and “u” for approximately three seconds at a 75 dB (±2 dB) intensity level. All voice samples were recorded and subsequently analyzed using the computer software PRAAT version 6.2, released in 2021. PRAAT is a free software package for speech analysis in phonetics that offers features such as waveform generation, wide and narrow-band spectrograms, intensity contour, and pitch tracks. Additionally, it supports speech synthesis, including articulatory synthesis, and allows for sound recording, editing, and extraction of individual sounds for in-depth analysis, providing valuable information about pitch, intensity, formants, and more.

Statistical analysis

All data were recorded in Microsoft Excel (Microsoft Corp., Redmond, WA, USA) worksheets, and statistical analyses were conducted using SPSS version 29.0 (IBM Corp., Armonk, NY, USA). Categorical variables were presented as numbers and percentages, while continuous variables were expressed as mean ± standard deviation (SD). To test mean differences of the fundamental frequency, first formant, shimmer, and maximum phonation duration in the preoperative and postoperative periods, a paired t-test was employed. A p-value <0.05 was considered statistically significant.

## Results

A total of 41 patients were included in the study and all of them had normal laryngoscopic findings following thyroid surgery. The majority of the participants were female (85.4%) (Table 2).

**Table 2:** Demographic and clinical characteristics of the study population. Data are presented as n (%). Age is presented as mean (range).

Characteristics	Total (n = 41)	%
Gender		
Male	06	14.6
Female	35	85.4
Age (in years)	42.4 (23.0–66.0)	
Residence		
Urban	28	68.3
Rural	13	31.7
Profession		
Homemaker	28	68.3
Teacher	03	7.3
Student	02	4.9
Farmer	03	7.3
Driver	05	12.2
Histopathological diagnosis		
Benign colloid nodule	16	39.0
Multinodular goiter	07	17.2
Papillary carcinoma	11	26.8
Follicular carcinoma	06	14.6
Anaplastic carcinoma	01	2.4
Procedure performed		
Right hemithyroidectomy	10	24.4
Left hemithyroidectomy	05	12.2
Completion thyroidectomy	02	4.9
Total thyroidectomy	16	39.0
Total thyroidectomy with neck dissection	08	19.5
Subjective complaints of change in the quality of voice		
Present	01	2.4
Absent	40	97.6

The average age of the study population was 42.4 years, ranging from 23.0 to 66.0 years. Professions varied within the sample, with homemakers representing the largest group (68.3%), followed by drivers (12.2%) and teachers (7.3%). Histopathological diagnoses included benign colloid nodules (39.0%), papillary carcinoma (26.8%), multinodular goiters (17.2%), follicular carcinoma (14.6%), and anaplastic carcinoma (2.4%). Hemithyroidectomy was performed in 17 (41.4%) patients and total thyroidectomy in 24 (58.6%) patients, of whom eight patients underwent central compartment neck dissection. Completion thyroidectomies are included in the hemithyroidectomy group for the sake of convenience in statistical comparison. Except for one patient, the majority of participants reported no subjective change in their voice following surgery.

Table 3 provides a comprehensive comparison of preoperative and postoperative values for various voice parameters. All voice parameters exhibited a statistically significant change in the one-month postoperative period compared to preoperative values. The fundamental frequency decreased by 5.87%, the shimmer intensity slightly decreased by 1.37%, and the first formant frequency decreased by 6.24%. Moreover, the maximum phonatory duration showed a reduction of 4.35% from preoperative levels. Values of all four voice parameters improved at the three-month postoperative period and approached close to preoperative levels.

**Table 3:** Comparison of preoperative and postoperative values of various voice parameters. Values are expressed as mean ± SD. On paired t-test: degrees of freedom = 40. P-value <0.05 is significant.

Voice parameters	Preoperative, mean ± SD	One-month postoperative, mean ± SD	Percentage change	P-value	Three-month postoperative, mean ± SD	Percentage change	P-value
Fundamental frequency	238.01 ± 41.05	224.40 ± 42.14	5.87%	<0.01	234.75 ± 41.00	1.37%	<0.01
Shimmer	3.66 ± 0.43	3.71 ± 0.42	1.37%	0.0856	3.75 ± 0.41	2.46%	0.0003
First formant	648.84 ± 124.18	608.33 ± 118.82	6.24%	0.0006	628.59 ± 116.48	3.12%	0.0006
Maximum phonatory duration	18.64 ± 1.78	17.83 ± 1.89	4.35%	0.0020	18.60 ± 1.51	0.21%	0.7777

Table 4 and Table 5 outline a comparative analysis of the alterations in the key voice parameters from preoperative baseline values between the hemithyroidectomy and total thyroidectomy groups at the one-month and three-month postoperative intervals. On comparison of mean differences, fundamental frequency, first formant frequency, and shimmer showed no significant differences between the two groups (p > 0.05). However, the maximum phonation duration differed significantly between the two groups (p < 0.05), with slightly greater changes in the hemithyroidectomy group at both one-month and three-month postoperative periods.

**Table 4:** Comparison of change in parameters of voice among hemithyroidectomy vs. total thyroidectomy group in the one-month postoperative period. Values are expressed as mean ± SD. P-value <0.05 is considered significant.

Voice parameters	Hemithyroidectomy (n = 17) (mean difference)	Total thyroidectomy (n = 24) (mean difference)	P-value
Fundamental frequency	11.97 ± 10.03	14.78 ± 13.47	0.4724
Shimmer	0.0324 ± 0.1190	0.0471 ± 0.1691	0.7592
First formant	32.92 ± 48.47	45.88 ± 81.55	0.5285
Maximum phonatory duration	1.51 ± 2.07	0.32 ± 0.84	0.0364

**Table 5:** Comparison of change in parameters of voice among hemithyroidectomy vs. total thyroidectomy group in the three-month postoperative period. Values are expressed as mean ± SD. P-value <0.05 is considered significant.

Voice parameters	Hemithyroidectomy (n = 17) (mean difference)	Total thyroidectomy (n = 24) (mean difference)	P-value
Fundamental frequency	3.17 ± 2.99	3.33 ± 3.77	0.8792
Shimmer	0.05 ± 0.0538	0.1154 ± 0.1759	0.0977
First formant	16.46 ± 24.24	22.94 ± 40.78	0.5286
Maximum phonatory duration	0.41 ± 1.10	0.22 ± 0.69	0.0447

## Discussion

This study provides valuable insights into the postoperative changes in four key parameters of voice and the impact of the extent of thyroid surgery on voice.

The majority of the participants experienced no subjective change in their voice following surgery. However, on objective voice analysis, we found a significant change in all the parameters of voice, among which the fundamental frequency and first formant frequency were most affected. The fundamental frequency represents the baseline pitch of a voice, while the first formant frequency is related to the vertical position of the tongue and the degree of elevation of the laryngeal framework during speech production [8,9]. These two parameters play a significant role in defining vocal characteristics and are essential for understanding speech production and phonetic identity. The laryngeal framework is stabilized by four strap muscles of the neck. These strap muscles help regulate the position and tension of the larynx during the production of speech and swallowing. During thyroid surgery, the strap muscles (sternothyroid and sternohyoid) are often divided or manipulated to access the thyroid gland. This division can disrupt the normal biomechanics and support of the larynx, potentially leading to changes in fundamental frequency and first formant frequency. In their prospective cohort study, Barron et al. found no discrepancy in postoperative subjective voice outcomes following the division of the sternothyroid muscle [10]. However, their study did not include objective voice analysis and focused solely on patients’ self-reported experiences of voice changes post-thyroid surgery.

When comparing changes in voice parameters between patients who underwent hemithyroidectomy and those who underwent total thyroidectomy, significant alterations were observed in both fundamental frequency, shimmer intensity, and first formant frequency. These changes were more pronounced in patients who underwent total thyroidectomy, although no statistical significance was noted. This suggests that the extent of thyroid surgery may have a differential impact on vocal parameters, with total thyroidectomy potentially exerting a greater effect on fundamental and first formant frequencies compared to hemithyroidectomy. The compression effect from large thyroid tumors can alter the position and function of the vocal cords, leading to voice alterations [11]. Removal of these tumors relieves long-standing pressure effects on the larynx and cervical trachea, which may result in voice changes even in the absence of direct nerve injury. Several studies have observed a similar correlation between the extent of thyroid surgery and postoperative voice impairment [12-14]. Their studies indicate that voice alterations worsen to a statistically significant degree following total thyroidectomy compared to hemithyroidectomy. Additionally, Jain et al., employing multivariate analysis, demonstrated that both the weight and volume of the thyroid gland significantly influence changes in voice quality after surgical removal, particularly in cases of papillary carcinoma [14].

We also found a slight change in shimmer intensity among both the hemithyroidectomy and total thyroidectomy groups. Shimmer reflects the irregularity or instability in the vibratory pattern of the vocal folds during phonation [15]. Shimmer is typically calculated as the percentage difference between the peak-to-peak variations in amplitude of consecutive vocal cycles [16]. Higher shimmer values indicate greater variability in vocal fold vibration. Sinagra et al. performed voice analysis in patients who underwent total thyroidectomy and noted no significant change in shimmer values among smokers and non-smokers [3]. The probable reason for the change in shimmer intensity in our study may be attributed to undocumented cricothyroid muscle injury during dissection of the thyroid [17,18].

Limitations

Several limitations need consideration in interpreting the findings of this study. First, the study had a relatively short follow-up period of three months, which may not capture long-term changes in voice quality that could emerge over time. Additionally, the sample size of 41 patients is relatively small, which may limit the generalizability of the findings to a broader population. Moreover, the study focused solely on objective voice parameters and did not incorporate any subjective questionnaires of voice quality.

## Conclusions

Our study on post-thyroidectomy patients with intact laryngeal nerves revealed that fundamental frequency and first formant frequency were significantly affected by the extent of thyroid surgery, with the total thyroidectomy group showing higher values. Despite anatomically intact laryngeal nerves with subjectively normal voice, subtle changes in voice parameters were observed postoperatively. These findings emphasize the importance of considering the extent of thyroid surgery in assessing postoperative voice outcomes and highlight the need for further research on the factors influencing voice alterations beyond laryngeal nerve dysfunction.
